# Cystic bleeding of the pineal gland, correlation with psychotic onset and other associated symptoms? Case report and review of the literature

**DOI:** 10.1192/j.eurpsy.2026.12179

**Published:** 2026-07-31

**Authors:** D. Del Maestro, N. Fascendini, S. Fumagalli, D. Garino

**Affiliations:** Clinica Santa Croce, Orselina, Switzerland

## Abstract

**Introduction:**

The pineal gland is an epithalamic gland accountable for the secretion of the hormone Melatonin, responsible for the correct sleep-wake rhythm, as well as indirectly influencing the hormones GH and Leptin. In current literature, correlations with acute pathological conditions of the pineal gland and the emergence of symptoms reliable to a psychotic state, or any psychiatric disorder, are scarce and incomplete.

**Objectives:**

Our case illustrates the diagnostic difficulty and complexity in a 16-year-old patient whose psychiatric symptomatic presentation is not standard and where the problem of a differential diagnosis of organic etiology poses a further challenge in choosing the therapeutic direction.

**Methods:**

With this poster, we try to describe the case of an who came to our attention with a psychotic onset likely related to bleeding from a pineal gland cyst. Over the course of a year and a half, he underwent five hospitalizations at our facility, allowing us to observe the evolution of his pathological condition. Furthermore, we decided to present the current literature on the topic in order to obtain a comparison with our case.

**Results:**

The hieratic symptomatic origin at the neuropsychiatric level remains not entirely clear, although some studies suggest the role of the hormone melatonin as important in the context of psychotic development, in the development of high-risk states and in other mood disorders. Despite this, case reports in the literature regarding correlations with pineal gland dysfunction describe pathological conditions with non-canonical characteristics, overlapping with our case. Therefore, it is possible to hypothesize a similar etiopathogenesis.

**Conclusions:**

This case demonstrates how a correlation can actually exist between pineal gland disorders and psychiatric disorders, although the limited scientific literature on the subject currently does not provide clear indications on the management of such a case.

Further clinical and neurochemical research is needed to firmly establish a correlation between pineal gland disorders and the emergence of psychotic symptoms

**Disclosure of Interest:**

None Declared

